# Supplementary material for: Identification of hub genes associated with oxidative stress in heart failure and their correlation with immune infiltration using bioinformatics analysis
Source: PeerJ. 2023 Aug 18;11:e15893. doi: 10.7717/peerj.15893 (PMC10441528; doi:10.7717/peerj.15893)
Supplement: Supplemental Information 1 — (A). Images of heart ultrasound; (B). LVEF: left ventricular ejection fraction; Values are expressed as mean (±SD) (n = 5); ***P < 0.001 vs Sham group. [file peerj-11-15893-s001.docx]

**Supplementary materials 1**

**Figure S1. Study protocol.**


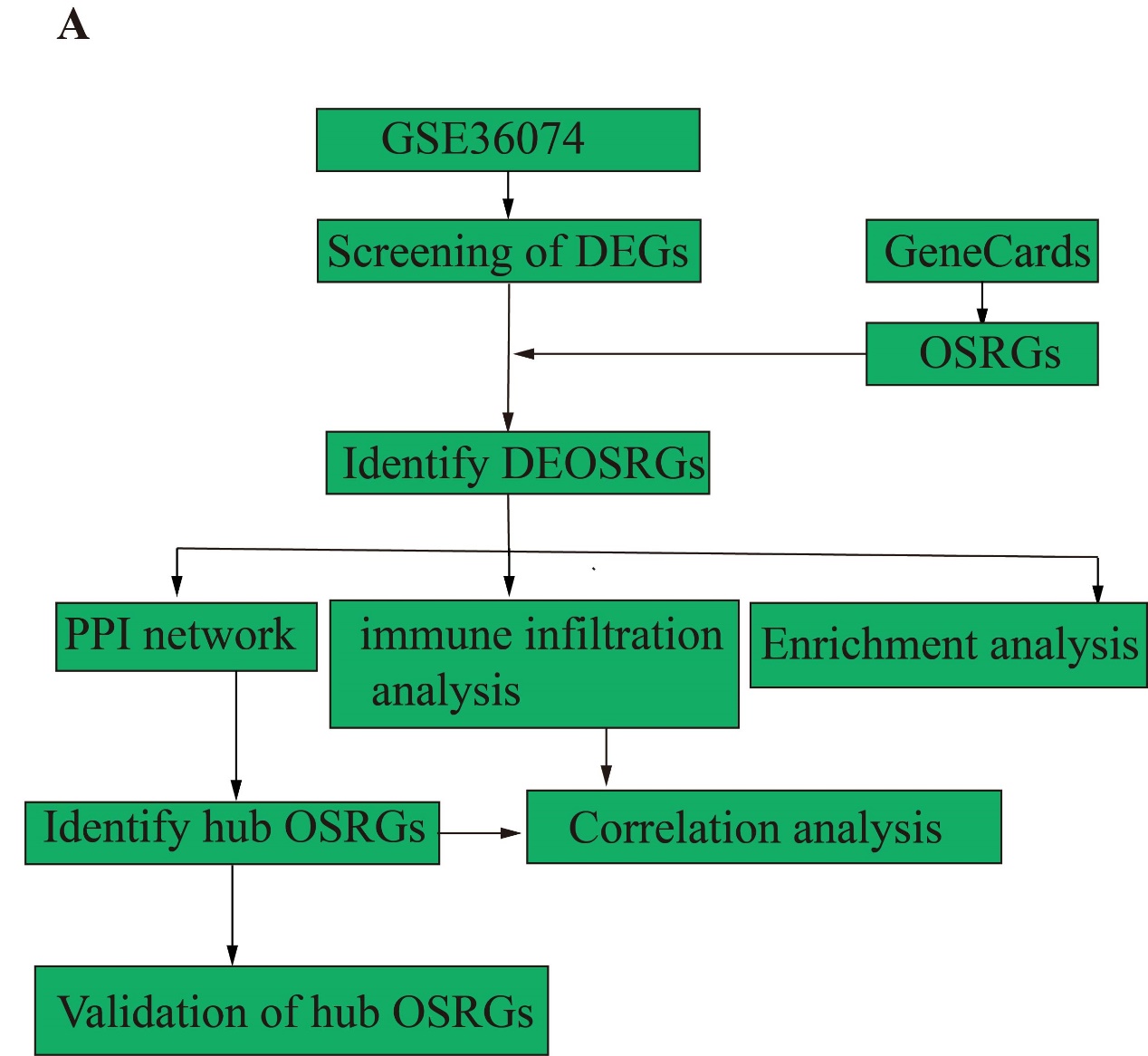


(A) Workflows of the study. ​Firstly, we downloaded the expression profile (GSE36074) to screen the DEGs. Then, Based on OSRGs, extracted from GeneCards websites, we obtained the OSRDEGs. Subsequently, we performed the functional enrichment analysis of OSRDEGs. We also constructed a PPI network to screen the hub genes. Furthermore, we analyzed the proportions of immune cells between the TAC and group samples and investigated their correlation with hub genes. Finally, we performed a TAC mouse model to validate the bioinformatics analysis results by RT-PCR, IHC and Western blot.

**Figure S2. Established TAC-induced mouse model of HF**


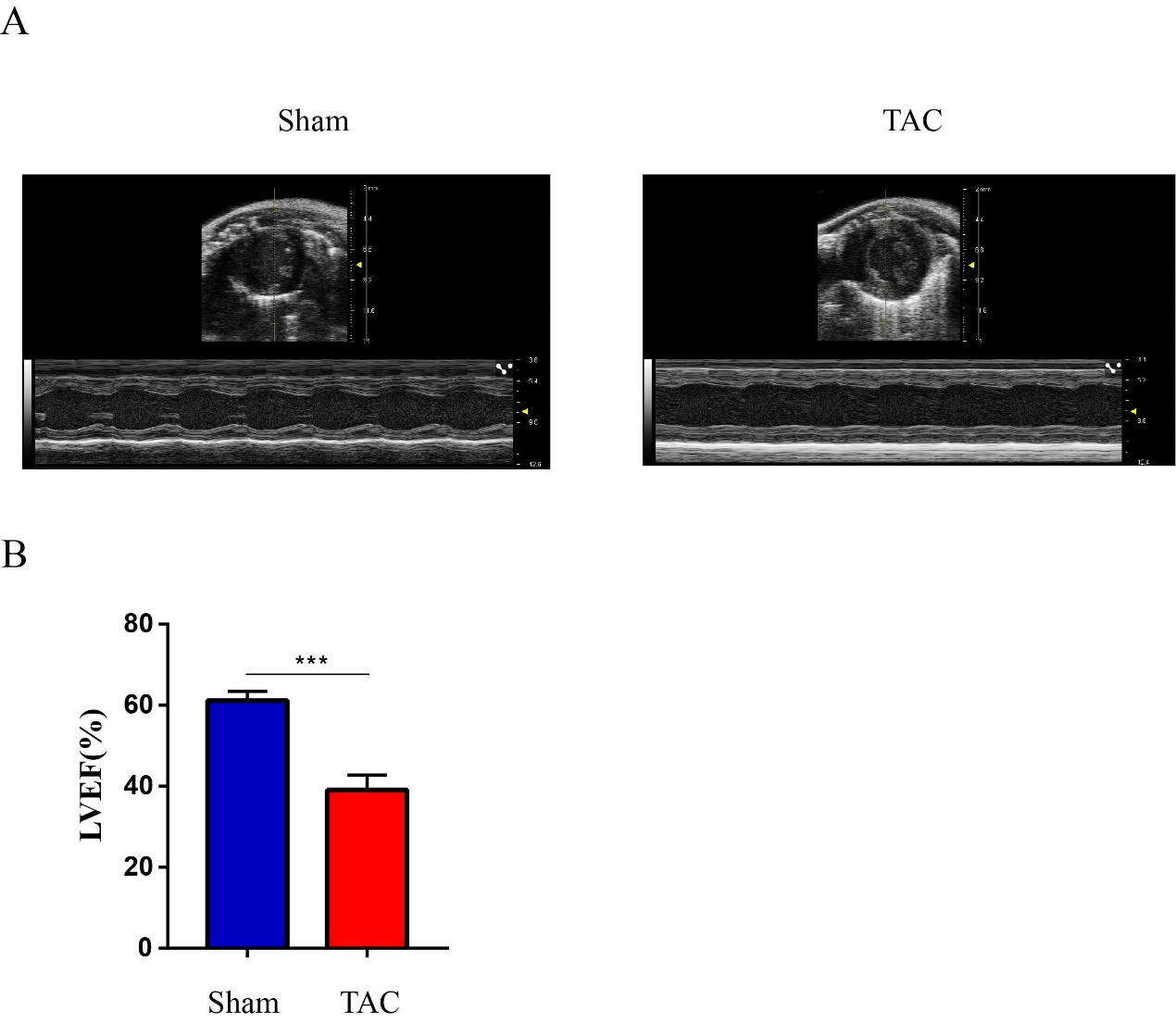


(A). Images of heart ultrasound; (B). LVEF: left ventricular ejection fraction; A two-tailed unpaired Student’s t-test was used to compare two groups. Values are expressed as mean ± SD (n=5); ****P* < 0.001vs Sham group.
